# Quantitative whole-body muscle MRI in idiopathic inflammatory myopathies including polymyositis with mitochondrial pathology: indications for a disease spectrum

**DOI:** 10.1007/s00415-024-12191-w

**Published:** 2024-03-05

**Authors:** Lea-Katharina Zierer, Steffen Naegel, Ilka Schneider, Thomas Kendzierski, Kathleen Kleeberg, Anna Katharina Koelsch, Leila Scholle, Christoph Schaefer, Arne Naegel, Stephan Zierz, Markus Otto, Gisela Stoltenburg-Didinger, Torsten Kraya, Dietrich Stoevesandt, Alexander Mensch

**Affiliations:** 1 Department of Neurology, University Medicine Halle, Ernst-Grube-Str. 40, 06120 Halle (Saale), Germany; 2 Department of Radiology, University Medicine Halle, Halle (Saale), Germany; 3 Department of Neurology, Alfried-Krupp-Krankenhaus Essen, Essen, Germany; 4 Department of Neurology, St. Georg Hospital Leipzig, Leipzig, Germany; 5 Department of Internal Medicine II, Rheumatology, University Medicine Halle, Halle (Saale), Germany; 6 Goethe Center for Scientific Computing (G-CSC), Goethe University, Frankfurt/Main, Germany; 7 Institute of Cell and Neurobiology, Charité University Medicine Berlin, Berlin, Germany

**Keywords:** qMRI, Myositis, PM-Mito, IBM, Inclusion body myositis, Imaging biomarker

## Abstract

**Objective:**

Inflammatory myopathies (IIM) include dermatomyositis (DM), sporadic inclusion body myositis (sIBM), immune-mediated necrotizing myopathy (IMNM), and overlap myositis (OLM)/antisynthetase syndrome (ASyS). There is also a rare variant termed polymyositis with mitochondrial pathology (PM-Mito), which is considered a sIBM precursor. There is no information regarding muscle MRI for this rare entity. The aim of this study was to compare MRI findings in IIM, including PM-Mito.

**Methods:**

This retrospective analysis included 41 patients (7 PM-Mito, 11 sIBM, 11 PM/ASyS/OLM, 12 IMNM) and 20 healthy controls. Pattern of muscle involvement was assessed by semiquantitative evaluation, while Dixon method was used to quantify muscular fat fraction.

**Results:**

The sIBM typical pattern affecting the lower extremities was not found in the majority of PM-Mito-patients. Intramuscular edema in sIBM and PM-Mito was limited to the lower extremities, whereas IMNM and PM/ASyS/OLM showed additional edema in the trunk. Quantitative assessment showed increased fat content in sIBM, with an intramuscular proximo-distal gradient. Similar changes were also found in a few PM-Mito- and PM/ASyS/OLM patients. In sIBM and PM-Mito, mean fat fraction of several muscles correlated with clinical involvement.

**Interpretation:**

As MRI findings in patients with PM-Mito relevantly differed from sIBM, the attribution of PM-Mito as sIBM precursor should be critically discussed. Some patients in PM/ASyS/OLM and PM-Mito group showed MR-morphologic features predominantly observed in sIBM, indicative of a spectrum from PM/ASyS/OLM toward sIBM. In some IIM subtypes, MRI may serve as a biomarker of disease severity.

**Supplementary Information:**

The online version contains supplementary material available at 10.1007/s00415-024-12191-w.

## Introduction

Idiopathic inflammatory myopathies (IIM) are rare and heterogeneous acquired autoimmune disorders. They present with a varying degree of muscular weakness, muscular inflammation, and a spectrum of extramuscular manifestations [1, 2]. The current classification includes dermatomyositis (DM), sporadic inclusion body myositis (sIBM), immune-mediated necrotizing myopathy (IMNM), and antisynthetase syndrome (ASyS)/overlap myositis (OLM) [3, 4]. There is a vivid debate as to polymyositis (PM) should be considered a distinct IIM entity, mainly based on clinicoseropathological studies showing that the majority of PM specimen can be classified into one of the other IIM categories after thorough re-evaluation [5, 6]. However, a small but relevant number of patients with histological presentation of IIM remain seronegative and thus may not be captured by the current classification [7].

This is exemplified by a rare IIM-variant termed polymyositis with mitochondrial pathology (PM-Mito). This disease was first described based on a cohort of 10 patients with clinical phenotype resembling sIBM, but without typical histopathological findings, such as vacuoles and protein aggregates. However, in addition to muscular inflammation, myohistology in all patients showed marked increase of cytochrome c oxidase (COX)-negative muscle fibers and multiple mtDNA deletions, suggesting a mitochondrial pathology [8]. Interestingly, COX-negative muscle fibers are also found in sIBM [9, 10]. Based on the clinical impression and histological overlap, the authors considered PM-Mito a variant of sIBM [8]. Since then, several studies have provided further evidence for a sIBM disease spectrum, including PM-Mito as precursor or slowly progressive variant [11–15]. However, others reported a relevant response to immunosuppressive treatment in a variable portion of PM-Mito patients, atypical for sIBM. In addition, some of the PM-Mito-patients did not develop an sIBM phenotype, even after several years of disease duration [16, 17]. Therefore, PM-Mito may comprise a heterogeneous subset of disease entities with varying potential for progression to sIBM.

While immunosuppressive therapy is generally considered effective in other forms of IIM, patients with sIBM typically do not show a sustained improvement [18], and initiation of immunosuppression may be detrimental, increasing drug-related morbidity [19, 20]. In this regard, sufficient and early delineation of the different IIM entities is of particular importance. While most patients with sIBM present with a characteristic clinical and histological phenotype, a significant number of patients are initially misdiagnosed [21–23]. One reason is the lack of specific features in early disease stages of sIBM [22]. Therefore, alternative diagnostic parameters for disease delineation and monitoring are mandatory. Muscle MRI has the potential to yield diagnostic specificity, and to provide an objective method to measure disease severity. While general information on MRI in IIM is limited, few studies have reported findings for specific IIM subtypes [24, 25].

In patients with PM/ASyS/OLM, the typical feature is a symmetrical diffuse edema, mainly affecting proximal muscle groups [26–28]. Degenerative pathology is initially negligible in most cases, but may be encountered to varying degrees in later stages [27]. While reports on muscle MRI in IMNM suggest a similar pattern of muscular edema, there appears to be an earlier and more pronounced muscular damage with fatty infiltration [29–31]. In sIBM, degenerative changes appear to be variable and potentially widespread. However, early and consistent degeneration of quadriceps femoris is frequently observed, with selective sparing of the rectus femoris and the adductor compartment [32–36]. In lower legs, the medial head of the gastrocnemius is predominantly affected [33–36]. Several authors reported an intramuscular gradient in sIBM, with distal parts of the muscle being more severely affected [32, 34, 35]. Furthermore, asymmetry is considered to be a common feature [32, 34]. Although muscular edema is common, it appears to be less prominent than in other IIMs [36]. Both semi-quantitative and quantitative evaluations of degenerative changes in sIBM were shown to correlate with disease severity [34, 36]. Moreover, a predefined sIBM-specific pattern of muscle involvement has been demonstrated to sufficiently differentiate sIBM from other IIM variants [37].

Although the value of the available MRI-studies in IIM is unequivocal, there are some obvious limitations and unsolved issues. Most studies do not rely on systematic semi-quantitative assessment of the individual muscles (by means of a grading system), impeding interpretation. Quantitative imaging strategies have only been used in very few studies. In most cases, exclusive imaging of the lower extremities instead of whole-body examination was used, resulting in a relevant lack of information. Of particular interest, no MRI findings in patients with PM-Mito have been reported so far, despite the ambiguity in terms of classification and delineation to other IIM variants. Therefore, the aim of the present study was a comprehensive semiquantitative and quantitative evaluation of whole-body muscle MRI findings in different IIM variants including PM-Mito.

## Methods

### Patients and control subjects

This retrospective study included all patients with histologically confirmed IIM who underwent whole-body muscle MRI as part of routine diagnostics at the Neuromuscular Center Halle by September 2020. MRIs, and histological and clinical data were anonymized for analysis. As control, 20 healthy volunteers (age 33–74 years, 10 men, 10 women), who underwent whole-body MRI as part of a prospective study to establish a standard for MRI quantification were included.

### Clinical data

Clinical data were obtained by careful review of the patient records. To account for clinical severity, a modified variant of the Medical Research Council sum score (MRC-SS) including finger flexion as a typical symptom of sIBM (modified MRC-SS) was used [38], resulting in a maximum MRC sum score of 70.

### Histologic reevaluation and assignment to IIM subgroups

All muscle biopsies (except for 2 biopsies which were performed at external hospitals) were re-evaluated by an experienced neuropathologist (GS-D) blinded to the clinical data. A standardized protocol was used, focusing on the presence of endomysial infiltrates, excessive necrosis, major histocompatibility complex (MHC-I) upregulation, rimmed vacuoles, sarcoplasmic protein aggregates, and mitochondrial pathology (i.e., COX-negative fibers exceeding age-related alterations).

After histopathological re-evaluation, assignment to a respective IIM subgroup was facilitated by a neurologist (AM) highly experienced in diagnosing and managing patients with IIM based on the available clinicoseropathological information. All patients fulfilling the 2011 ENMC diagnostic criteria for clinically (disease duration more than 12 months, onset after 45 years of age, knee extension weakness ≥ hip flexion weakness and/or finger flexion weakness > shoulder abduction weakness, serum CK less than 15 fold above the upper limit of normal) or clinico-pathologically (met clinical features and histologically confirmed endomysial inflammation, rimmed vacuoles and protein accumulations) defined sIBM were assigned to the sIBM subgroup [39, 40]. All patients with a relevant endomysial infiltrate, MHC-I-upregulation, and lack of sIBM-defining clinical and histological features were suspected of having ASyS or OLM. Due to the lack of structured serological information, and in light of the aforementioned controversies regarding the terminology of IIM, it was decided to also include “seronegative” IIM patients. These patients were pooled in the Polymyositis/Anti-Synthetase-Syndrome/Overlap-Myositis subgroup (PM/ASyS/OLM). However, separate information on the IIM variants (PM/unspecific myositis, ASyS and OLM) of this pooled group can be found in Suppl. Tab. 5. Patients fulfilling the above-mentioned criteria for PM, and additionally displaying a relevant mitochondrial pathology (age-exceeding COX-negative muscle fibers) in myohistology were classified in the PM-Mito subgroup [8, 12, 15, 16]. Diagnosis of IMNM was based on histological appearance with predominant myonecrosis in different stages and MHC-I-upregulation [41].

### MRI acquisition

Whole-body MR imaging was performed using a 3 T MRI (Skyra; Siemens, Erlangen, Germany) with two flexible 18-channel transmit/receive surface body coils for neck, thorax, arms, abdomen and pelvis, and a 36-channel angiography coil for the legs. All patients were scanned in supine position. Imaging included a T1-weighted Dixon Turbo Spin Echo sequence (TSE) in axial plane with 8 mm slice thickness. No sedation or contrast agent was used.

### Semiquantitative grading

MRIs were reviewed by two independent evaluators blinded to the clinical data and diagnosis, one being an experienced radiologist with neuromuscular focus (DS), the other doctoral researcher extensively trained in muscle imaging (L-KZ). As previously established in this setting [42], fatty fibrous degeneration was graded using the five-point semi-quantitative grading scale established by Fischer et al. [43]. Briefly, stage 0 refers to a normal appearance, while stages 1–4 show an increased T1-signal intensity to varying degrees (1—slightly, non-confluencing; 2— mildly confluencing, less than 50% of muscle; 3—confluencing in more than 50% of muscle; 4—replacement of entire muscle). In view of the expectation of an intramuscular heterogeneity in the degree of fatty fibrous degeneration [34], the highest observed Fischer grade per muscle was assigned, same applied in case of asymmetry. Due to strong intramuscular differences, the gluteus minimus was analyzed separately in the ventral and dorsal part. Intramuscular edema was graded dichotomously (presence or absence) based on an increase in signal intensity in the water-only image obtained by the Dixon technique.

### Quantitative MRI evaluation

Muscles that appeared to provide a good separation between IIM subgroups were selected for quantification. The absolute fat content of 12 muscles (Table 2) was determined using the method described by Dixon [44]. Pre-defined anatomical landmarks were used as a reference to identify the region of interest (Suppl. Tab. 1). Measurements were made in proximal, mid and distal parts of each muscle. The flexor hallucis longus is considerably short and therefore allowed only two measurements (proximal, distal). For the same reason, fat fraction of gluteal and axial muscles was determined at a single site.

### Statistical analysis

The focus of this work is on description. However, for the sake of completeness, statistics were also calculated to identify significant differences. Statistical analyses were performed using IBM SPSS Statistics, version 25 (IBM, Armonk, NY, USA) and JASP (JASP Team, 2023, Version 0.17.1). Due to the small group sizes, normal distribution could not be assumed based on the central limit theorem. Differences between groups were assessed using the Kruskal–Wallis test. Post hoc comparisons were performed using the Dunn test with Bonferroni correction. Significance of correlations was tested using Pearson's and Spearman's correlations. The detailed results of statistical analyses are summarized in Suppl. Tab. 7.

## Results

### Patients

Clinical characteristics of the patients are summarized in Table 1. Nine patients fulfilled the ENMC criteria of sIBM, whereas two patients were too young according to ENMC (40 and 41 years). However, due to their clear clinical and histological phenotype, these were included in the sIBM group. This group accordingly comprised 11 patients. Seven patients were assigned to the PM-Mito group, while 11 patients fulfilled the criteria for PM/ASyS/OLM, and 12 patients those for IMNM.

**Table 1:** Clinical characteristics of the studied patients

		sIBM	PM-Mito	PM/ASyS/OLM	IMNM	control
No. of subjects		11	7	11	12	20
F/M		3/8	6/1	7/4	7/5	10/10
Age at MRI	Median (Range)	58 (40–75)	64 (33–67)	65 (45–88)	72 (48–77)	55 (33–74)
BMI	Median (Range)	26 (22–41)	25 (18–33)	26 (16–35)	26 (20–37)	26 (22–32)
Disease duration (mo)	Median (Range)	36 (6–180)	36 (24–192)	12 (0–72)	11 (2–180)	–
Laboratory findings						
CK max. (fold ULN)	Median (Range)	4 (1.5–21)	6 (N-12)	13.6 (N-33)	32.6 (N-46)	–
CK at MRI (fold ULN)	Median (Range)	3.5 (N-8)	2.5 (N-12)	1.7 (N-20)	12.4 (N-46)	–
MSA-positive	No. (percent)	7 (64%)	3 (43%)	6 (55%)	10 (83%)	–
5NT1A (MUP44)	No.	6	2		1	–
Ro	No.	3	–	3	3	–
PL12	No.	1	–		–	–
Mi-2	No.	–	–	1	–	–
NXP2	No.	–	–	1	–	–
Jo-1	No.	–	–	2	–	–
Ku	No.	–	–	1	–	–
SnRNP	No.	–	–	1	–	–
PmScl	No.	–	–	3	1	–
SRP	No.	–	1	1	6	–
HMGCR	No.	–	–	–	4	–
Immunosuppression						
IST at MRI	No. (percent)	5 (45%)	3 (43%)	8 (73%)	8 (67%)	–
GC	No.	3	2	6	6	–
AZA	No.	1	2	3	1	–
MTX	No.	–	–	2	3	–
MMF	No.	1	–	1	–	–
RTX	No.	–	–	1	–	–
IVIG	No.	1	–	1	–	–
Clinical examination						
Dysphagia	No. (percent)	7 (64%)	0	2 (18%)	4 (33%)	–
Dysarthria	No. (percent)	2 (18%)	0	2 (18%)	2 (17%)	–
Myalgia	No. (percent)	3 (27%)	3 (43%)	7 (64%)	5 (42%)	–
Proximal UL weakness	No. (percent)	9 (82%)	4 (57%)	8 (73%)	10 (83%)	–
Distal UL weakness	No. (percent)	11 (100%)	2 (29%)	2 (18%)	4 (33%)	–
Proximal LL weakness	No. (percent)	10 (91%)	5 (71%)	9 (82%)	12 (100%)	–
Distal LL weakness	No. (percent)	5 (45%)	2 (29%)	0	2 (17%)	–
Modified MRC-SS	Median (Range)	57 (43–69)	66 (59–70)	64 (60–70)	62 (53–65)	–
Histology						
Endom. Infl	No. (percent)	11 (100%)	7 (100%)	6 (67%)^c^	5 (42%)	–
RV	No. (percent)	11 (100%)	3 (43%)^a^	3 (33%)^c^	3 (25%)	–
Protein acc	No. (percent)	11 (100%)	1 (14%)^b^	3 (33%)^c^	2 (17%)	–
MHC-I	No. (percent)	11 (100%)	7 (100%)	4 (44%)^c^	4 (33%)	–
COX-neg	No. (percent)	9 (82%)	7 (100%)	0	0	–

*No.* number, *F/M* female/male, *BMI* Body Mass Index, *mo* months, *CK* Creatine Kinase, *max.* maximum, *ULN* Upper limit of normal, *N* normal*, MSA* myositis-specific antibodies, *IST* Immunosuppressive treatment, *GC* Glucocorticoids, *AZA* Azathioprine, *MTX* Methotrexate, *MMF* Mycophenolate mofetil, *RTX* Rituximab, *IVIG* Intravenous Immunoglobulins, *UL* Upper limb, *LL* Lower limb, *MRC-SS* Medical Research Council Sum Score, *Endom. Infl.* Endomysial inflammation, *RV* Rimmed vacuoles, *acc.* accumulation, *neg.* negative

^a^In 1 fiber

^b^Weak positivity in 1 fiber

^c^*N* = 9 (2 external biopsies were not available for reevaluation);

The maximum CK levels measured during the course of the disease were higher in IMNM (median 32.6 × ULN) and PM/ASyS/OLM (median 13.6 × ULN) than in sIBM (median 4 × ULN), and PM-Mito (median 6 × ULN). The proportion of patients with myositis-specific autoantibodies (MSA) varied between groups, being most prevalent in the IMNM group (83%), and detected in about half of the patients in the other groups. At time of MRI, nearly half of the sIBM (45%) and PM-Mito (43%) patients received immunosuppressive treatment, while a higher proportion of the patients in PM/ASyS/OLM (73%) and IMNM (67%) groups were on treatment.

In terms of clinical presentation, patients with sIBM tended to be more severely affected (median MRC-SS 57), compared to PM-Mito (66), PM/ASyS/OLM (64), and IMNM (62). Dysphagia and dysarthria were prominent features in sIBM, whereas they were rare in PM/ASyS/OLM and IMNM, and absent in PM-Mito. In upper extremities, proximal weakness was present to a varying degree in all IIM subgroups, while distal muscle involvement was predominant in sIBM (100%), though also observed in IMNM (33%), PM-Mito (29%) and PM/ASyS/OLM (18%). Proximal lower limb weakness was a common feature of all IIM subgroups. Again, distal involvement was more common in sIBM, compared to the other IIM patients.

### Semi-quantitative analysis of fatty degeneration and pattern evaluation

**Fig. 1:**
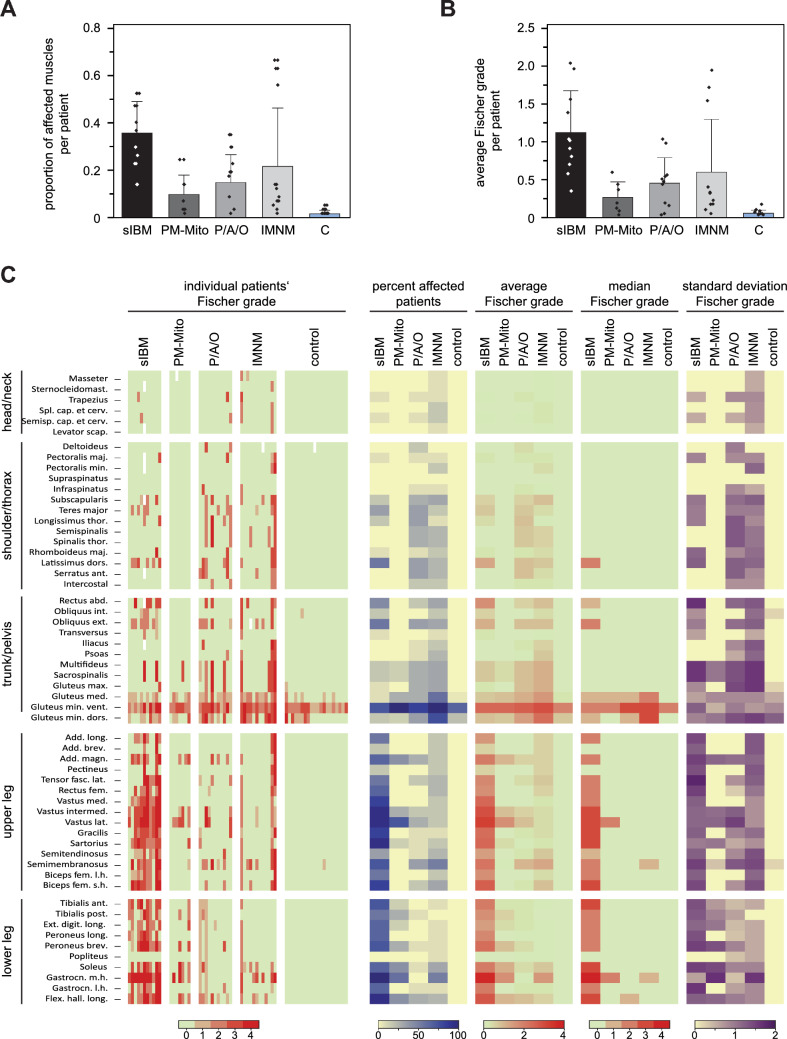
Semiquantitative evaluation of muscular degeneration in IIM. **A** Proportion of affected muscles per patient. Affection was defined as Fischer grade ≥ 2. Bars represent the average fraction of affected muscles, whiskers show one standard deviation, and dots account for the individual patient. Patients with sIBM show a significantly higher affection compared to PM-Mito and PM/ASyS/OLM (P/A/O). Note the high variability in IMNM. **B** Average Fischer score per patient. Bars represent the average Fischer grade calculated from all muscles, whiskers show one standard deviation, and dots account for the individual patient. Again, a higher average Fischer grade is seen in sIBM, while the IMNM group shows a high variability. **C** Individual and pooled results of the Fischer grades for all studied muscles. Heat map on the left shows the individual Fischer grades for each patient, stratified by subgroup. Heat maps on the right represent statistical indicators derived from each subgroup. A predominant lower limb involvement is seen in sIBM. There is no specific pattern in PM-Mito patients, while PM/ASyS/OLM and IMNM tend to affect proximal muscle groups (trunk/thorax). Note the consistent affection of the ventral parts of gluteus minimus in all groups (including control), suggestive of non-specificity. *Sternocleidomast.* Sternocleidomastoideus*, Spl.* Splenius*, cap.* capitis*, cerv.* cervicis*, Semisp.* Semispinalis*, scap.* scapulae*, maj.* major*, min.* minor*, thor.* thoracis*, dors.* dorsi/dorsal*, ant.* anterior*, abd.* abdominis*, int.* internus*, ext.* externus*, max.* maximus*, med.* medius/medialis*, min.* minimus*, vent.* ventral*, Add.* Adductor*, long.* longus/longum*, brev.* brevis*, magn.* magnus*, fasc. lat.* fasciae latae*, fem.* femoris*, intermed.* intermedius*, lat.* lateralis*, l.h.* long head*, s.h.* short head*, post.* posterior*, Ext. digit.* Extensor digitorum*, Gastrocn.* Gastrocnemius*, m.h.* medial head*, l.h.* lateral head, *Flex. hall.* Flexor hallucis longus

Fatty degeneration was present in all groups of IIM (Fig. 1A, Suppl. Tab. 2). However, whereas a small fraction of muscles was affected in PM-Mito (median 7%, range 2–25%) and PM/ASyS/OLM (18%, 0–35%; no differences between PM, ASyS, and OLM, Suppl. Tab. 5), widespread fatty degeneration was observed in sIBM (37%, 14–53%). There were mixed results in IMNM, with some patients showing extensive muscular involvement, especially after longer disease duration (11%, 2–67%). Similar findings were seen with regard to severity of fatty infiltration (Fig. 1B, Suppl. Tab. 2). The average Fischer grade appeared to be higher in sIBM patients (1.12, range 0.35–2.04), compared to PM-Mito (0.26, range 0.03–0.59) and PM/ASyS/OLM (0.42, range 0.03–1.03) patients. Again, heterogeneous results were observed for IMNM (0.59, range 0.05–1.94).

There were apparent differences in the pattern of muscular involvement (Fig. 1C*, *Suppl. Tab. 3). In sIBM, lower extremities were predominantly affected. In the thighs, the most prominent and consistent changes were seen in quadriceps femoris and sartorius, followed by hamstrings (with relative sparing of the semitendinosus and the short head of the biceps femoris), whereas adductors appeared to be less affected. In the lower legs, all muscles except popliteus showed marked fatty degeneration, most prominent in the medial head of the gastrocnemius and the flexor hallucis longus. Patients with PM-Mito showed a comparable modest fatty degeneration in most muscles. In some patients, there was relevant involvement of vastus lateralis and the medial head of gastrocnemius. In PM/ASyS/OLM and IMNM, there was no specific pattern of muscular affection. Proximal muscle groups, such as gluteal and paraspinal muscles, appeared to be more frequently affected. In IMNM, semimembranosus and the medial head of gastrocnemius were preferentially involved. However, as in PM-Mito, there was a high variability. A consistent involvement of the ventral parts of gluteus minimus was found in all groups of IIM, as well as in the control group, suggesting non-specificity.

### Muscular edema

**Fig. 2:**
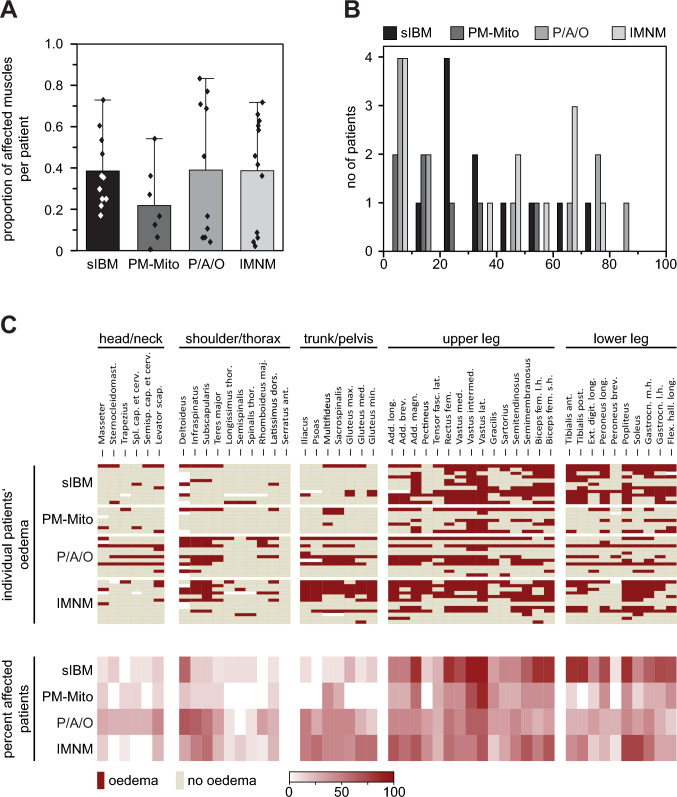
Distribution and severity of edema in IIM. **A** Proportion of affected muscles per patient. Bars represent the average percentage of affected muscles, whiskers show one standard deviation, and dots account for the individual patient. There is no significant difference in the number of edema-positive muscles between IIM subgroups. **B** Histogram plot showing the severity of edema as indicated by edema-positive muscles. There appears to be a tendency for a higher proportion of edema-positive muscles in a subset of PM/ASyS/OLM- and IMNM patients. **C** Individual and summarized evaluation of edema in different IIM subgroups. Upper part of the heat map shows the individual edema distribution of each patient, stratified by the IIM subgroup. Lower part of the heat map displays the proportion of patients positive for edema in the individual muscle. A predominant affection of the lower limbs is found in sIBM, while there is only moderate affection in PM-Mito. PM/ASyS/OLM (P/A/O) and IMNM subgroups show a pronounced affection of head/neck (P/A/O), shoulder/thorax, and trunk. *no* number, *Sternocleidomast.* Sternocleidomastoideus*, Spl.* Splenius*, cap.* capitis*, cerv.* cervicis*, Semisp.* Semispinalis*, scap.* scapulae*, thor.* thoracis*, maj.* major*, dors.* dorsi*, ant.* anterior*, max.* maximus*, med.* medius/medialis*, min.* minimus*, Add.* Adductor*, long.* longus/longum, *brev.* brevis*, magn.* magnus*, fasc. lat.* fasciae latae*, fem.* femoris*, intermed.* intermedius*, lat.* lateralis*, l.h.* long head*, s.h.* short head*, post.* posterior*, Ext. digit.* Extensor digitorum*, Gastrocn.* Gastrocnemius, *m.h.* medial head*, l.h.* lateral head, *Flex. hall.* Flexor hallucis longus

Overall, the average number of muscles affected by edema did not significantly differ between sIBM, PM/ASyS/OLM, and IMNM (Fig. 2A). However, analysis of distribution identified a subpopulation of PM/ASyS/OLM- and IMNM patients with a higher percentage of edema-positive muscles (Fig. 2B). Regarding the pattern of edema distribution (Fig. 2C, Suppl. Tab. 3), sIBM showed a predominant affection of the lower extremities, similar in thighs and lower legs. In PM/ASyS/OLM and IMNM, proximal muscle groups were more likely to be affected, including gluteal, axial and shoulder muscles. While lower legs were less commonly affected in PM/ASyS/OLM, a relevant involvement of triceps surae was seen in IMNM. However, distribution of edema—if present—appeared to be similar. In the PM/ASyS/OLM group, patients classified as having OLM tended to have less edema, whereas patients with PM had a higher percentage of muscles affected (Suppl. Tab. 5). In the PM-Mito subgroup, edema—if present—was predominantly found in the thighs.

### Asymmetry

Asymmetry of individual muscles is visualized in Suppl. Fig. 1A. Asymmetric fatty degeneration was a common phenomenon. However, the rate of asymmetry in Fischer grading was not significantly altered in the individual IIM subgroups (Suppl. Fig. 1B). There was a high inter-individual variety, especially in PM/ASyS/OLM- and IMNM patients. Regarding intramuscular edema, the PM/ASyS/OLM subgroup appeared to show a tendency toward symmetric involvement, while no differences were found in the other IIM subgroups (Suppl. Fig. 1C).

### Quantitative determination of mean fat fraction and proximo-distal gradient

**Fig. 3:**
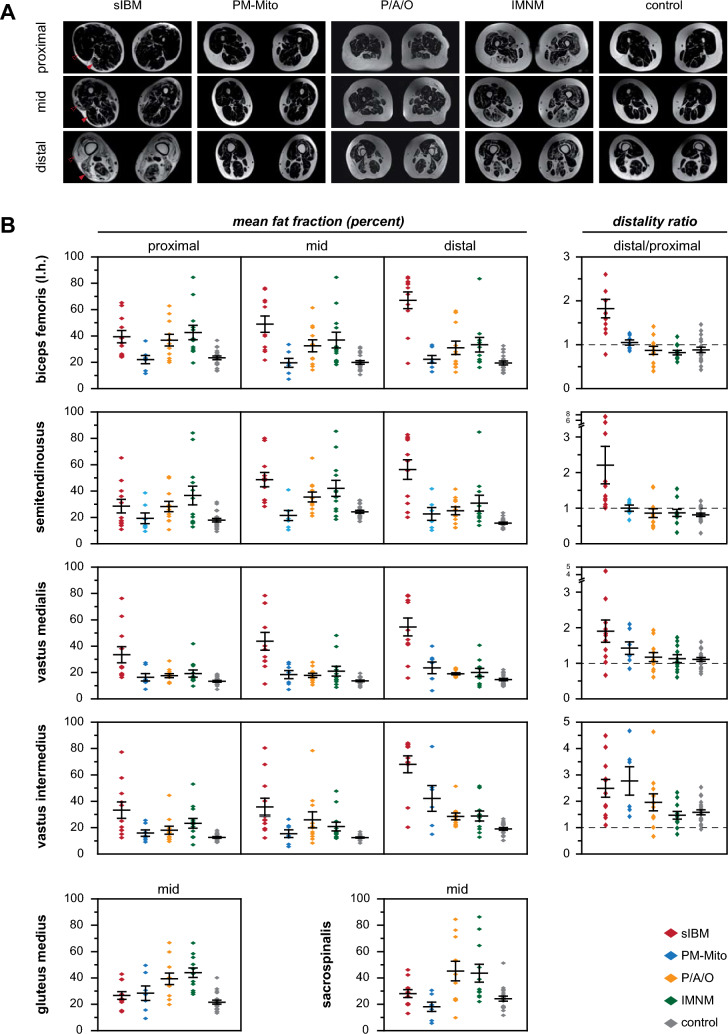
Quantitative assessment of muscular fat fraction in IIM subgroups. **A** Representative example images used to quantify the muscular fat fraction (MFF). Note the proximo-distal gradient of muscular degeneration in sIBM (red arrowhead). **B** MFF of selected muscles in proximal, mid, and distal parts of the respective muscle (left panels), and determination of the distality ratio (MFF of distal versus proximal parts, right panel). Middle dash represents the average MFF, while whiskers indicate one standard deviation, and dots show individual patient's values. Further information regarding the additional muscles studied can be found in Table 2 and Suppl. Fig. 2. MFF of sIBM patients is consistently elevated in all studied lower extremity muscles. While MFF in proximal muscle parts appears to be comparable to other IIM subtypes, there is a significantly higher MFF in distal parts of sIBM patients, resulting in an increased distality ratio. In sIBM, this proximodistal gradient is found in all studied leg muscles except for rectus femoris (shown in Suppl. Fig. 2). In some muscles, such as the vastus medialis and vastus intermedius, similar changes are present in a proportion of PM-Mito and PM/ASyS/OLM (P/A/O) patients. For additional examples see Suppl. Fig. 2. Note the relevantly increased MFF of gluteus medius and sacrospinalis in PM/ASyS/OLM and IMNM. *l.h.* long head

Representative sample images used for quantification are shown in Fig. 3A. In accordance with the semi-quantitative analysis, all studied muscles of the lower extremities showed a significantly higher mean fat fraction (MFF) in the sIBM group, compared to all other IIM groups (summary in Table 2, detailed information in Suppl. Tab. 4).

**Table 2:** Results of MRI quantification

		sIBM	PM-Mito	PM/ASyS/OLM	IMNM	control	*p* value
SAC							
* M*	Median (Range)	28.4 (13.0–46.1)	19.3 (5.6–30.6)	42.8 (9.8–84.5)	31.4 (22.0–86.3)	23.8 (11.6–51.3)	0.002
GLM							
* M*	Median (Range)	24.1 (14.4–42.9)	27.4 (9.2–49.5)	39.6 (19.8–66.7)	45.1 (27.6–66.5)	20.1 (13.3–40.1)	< 0.001
RF							
* P*	Median (Range)	43.4 (15.6–85.6)	15.1 (11.9–22.3)	19.8 (8.7–31.0)	24.1 (9.9–75.4)	14.1 (8.6–30.3)	< 0.001
* M*	Median (Range)	47.4 (15.5–83.0)	15.5 (7.0–24.5)	17.2 (9.4–37.6)	18.1 (9.4–73.4)	13.0 (6.7–22.7)	< 0.001
* D*	Median (Range)	44.6 (8.8–85.6)	8.2 (4.9–21.0)	13.3 (3.5–55.2)	14.1 (6.6–82.5)	9.4 (4.5–14.8)	< 0.001
* D/P*	Median (Range)	0.9 (0.4–1.3)	0.7 (0.4–1.0)	0.6 (0.3–4.4)	0.5 (0.2–1.5)	0.7 (0.3–1.1)	0.264
VM							
* P*	Median (Range)	23.8 (16.3–76.3)	13.8 (7.3–27.4)	16.6 (11.8–28.8)	15.0 (9.7–41.8)	13.1 (7.2–18.8)	< 0.001
* M*	Median (Range)	37.5 (11.3–78.4)	20 (7.1–27.7)	17.2 (10.6–27.8)	17.6 (8.8–48.1)	13.0 (8.7–19.4)	< 0.001
* D*	Median (Range)	52.1 (15.8–78.6)	27.3 (6.2–40)	17.7 (16.2–23.5)	17.9 (9.0–40.7)	13.9 (9.4–22.2)	< 0.001
* D/P*	Median (Range)	1.8 (0.7–4.5)	1.2 (0.8–2.1)	1.1 (0.6–1.9)	1.0 (0.6–1.7)	1.1 (0.7–1.6)	0.033
VI							
* P*	Median (Range)	25 (12.4–77.3)	13.7 (9.1–25.6)	16.2 (9.5–44.5)	21.8 (7.0–53.0)	12.3 (8.8–18.0)	< 0.001
* M*	Median (Range)	26 (12.2–80.4)	13.8 (5.6–26.4)	19.8 (8.3–78.4)	16.3 (11.9–47.7)	12.4 (8.3–16.9)	< 0.001
* D*	Median (Range)	72.8 (20.3–84.1)	42.2 (15.0–81.5)	26.4 (20.7–51.4)	27.6 (12.8–51.5)	18.9 (10.3–26.7)	< 0.001
* D/P*	Median (Range)	2.3 (1.1–4.5)	2.6 (1.4–4.7)	1.7 (0.6–4.6)	1.4 (0.7–2.3)	1.5 (0.9–2.5)	0.024
VL							
* P*	Median (Range)	22.5 (11.4–77.0)	12.4 (7.7–27.6)	16.5 (10.1–39.2)	17.6 (8.6–57.8)	12.4 (9.5–23.2)	0.003
* M*	Median (Range)	25.5 (12.3–77.4)	11.1 (5.1–35.4)	19.7 (11.0–68.8)	22.9 (7.8–52.8)	13.3 (8.5–18.4)	< 0.001
* D*	Median (Range)	79.7 (21.8–85.4)	24.8 (17.1–61.5)	25.7 (15.5–60.8)	19.2 (4.9–55.8)	19.0 (9.6–32.8)	< 0.001
* D/P*	Median (Range)	2.0 (1.0–4.7)	1.9 (0.9–6.8)	1.6 (0.5–3.8)	1.0 (0.6–2.3)	1.5 (0.8–2.9)	0.008
GR							
* P*	Median (Range)	34.8 (9.9–67.2)	10.6 (5.6–31.8)	15.5 (7.8–26.6)	17.7 (8.5–80.9)	11.6 (6.6–20.9)	< 0.001
* M*	Median (Range)	48.4 (12.9–76.9)	19.7 (6.4–39.6)	18.3 (7.5–37.2)	19.6 (10.5–62.4)	16.6 (8.2–27.7)	< 0.001
* D*	Median (Range)	78.1 (14.5–81.0)	19.6 (9.7–34.4)	18.2 (9.3–32.3)	19.1 (9.6–31.9)	15.0 (9.2–26.7)	< 0.001
* D/P*	Median (Range)	2.1 (0.9–5.4)	1.5 (1.0–2.1)	1.1 (0.5–1.9)	1.1 (0.3–1.6)	1.4 (0.9–2.3)	0.023
SAR							
* P*	Median (Range)	40.7 (16.4–69.2)	14.2 (9.2–43.3)	18.3 (4.9–34.4)	17.6 (11.8–37.6)	15.8 (11.3–26.1)	< 0.001
* M*	Median (Range)	58.2 (19.0–84.7)	20 (10.3–45.3)	26.6 (11.7–42.9)	24.0 (11.6–58.6)	22.2 (11.3–38.5)	0.001
* D*	Median (Range)	74 (17.2–83.6)	24.2 (8.8–72.2)	17.7 (11.4–67.1)	19.1 (8.4–37.9)	17.7 (7.6–30.8)	< 0.001
* D/P*	Median (Range)	1.6 (0.8–4.5)	1.2 (0.7–7.8)	1.1 (0.6–2.6)	1.0 (0.6–1.7)	1.1 (0.6–2.3)	0.15
ST							
* P*	Median (Range)	19.8 (10.9–65.2)	13.9 (9.4–38.6)	25.9 (10.7–50.8)	28.5 (12.8–84.1)	16.8 (9.2–31.6)	0.036
* M*	Median (Range)	48.1 (28.3–80.1)	19.1 (10.5–40.9)	31.7 (21.1–65.0)	37.4 (18.5–85.4)	23.2 (16.8–33.1)	< 0.001
* D*	Median (Range)	62.8 (20.1–82.8)	21 (10.2–41.7)	26 (12.1–41.5)	23.1 (13.9–84.8)	15.4 (10.8–23.5)	< 0.001
* D/P*	Median (Range)	1.5 (1.2–7.3)	1.2 (0.8–1.4)	0.9 (0.5–1.8)	1.0 (0.4–1.8)	0.9 (0.3–1.4)	< 0.001
BFLH							
* P*	Median (Range)	34.6 (24.0–65.2)	22.6 (11.5–36.2)	34.1 (20.0–62.8)	37.3 (19.5–84.5)	23.3 (13.4–36.6)	< 0.001
* M*	Median (Range)	40.8 (21.7–76.3)	19.3 (7.2–33.5)	33.3 (14.2–61.4)	29.1 (18.2–84.5)	18.7 (10.6–31.5)	< 0.001
* D*	Median (Range)	76.5 (19.2–84.7)	20.1 (12.8–33.1)	26.3 (12.5–58.9)	31.4 (16.1–83.4)	16.9 (11.6–32.6)	< 0.001
* D/P*	Median (Range)	1.7 (0.8–3.3)	1.0 (0.8–1.2)	0.9 (0.4–1.4)	0.8 (0.6 -1.2)	0.8 (0.4–1.4)	< 0.001
GMH							
* P*	Median (Range)	64.6 (14.5–79.9)	20.1 (7.7–66.5)	20 (13.0–53.1)	27.1 (7.5–73.3)	15.0 (8.3–20.0)	< 0.001
* M*	Median (Range)	74.1 (25.5–82.5)	25.1 (8.8–77.7)	26.4 (12.1–44.4)	25.5 (6.8–84.8)	17.1 (10.0–25.8)	< 0.001
* D*	Median (Range)	76.6 (61.0–85.5)	45.4 (81–82.3)	28.8 (15.3–63.3)	34.2 (5.2–83.4)	13.9 (5.8–37.4)	< 0.001
* D/P*	Median (Range)	1.2 (0.9–4.7)	1.1 (0.7–2.7)	1.3 (0.7–1.7)	1.0 (0.5–2.0)	1.0 (0.5–1.9)	0.066
FHL							
* P*	Median (Range)	35.9 (11.9–78.7)	21.1 (7.2–37.2)	31.4 (16.7–56.8)	28.4 (14.8–51.8)	18.4 (10.6–28.6)	0.006
* D*	Median (Range)	73.2 (33.6–83.1)	25.5 (12.1–71.1)	30.7 (15.4–53.8)	22.0 (7.4–51.1)	16.3 (9.5–36.6)	< 0.001
* D/P*	Median (Range)	1.7 (1.0–6.6)	1.6 (0.7–3.7)	1.1 (0.5–1.4)	0.9 (0.4–1.7)	0.9 (0.6–1.3)	0.002

*SAC* Sacrospinalis, *GLM* Gluteus medius, *RF* rectus femoris, *VM* Vastus medialis, *VI* Vastus intermedius, *VL* Vastus lateralis, *GR* Gracilis, *SAR* Sartorius, *ST* Semitendinosus, *BFLH* Biceps femoris long head, *GMH* Gastrocnemius medial head, *FHL* Flexor hallucis longus, *P* proximal, *M* mid, *D* distal

In sIBM patients, a remarkable intramuscular gradient was present in most muscles. While proximal parts of the muscle showed a MFF comparable to other IIM patients or controls, distal muscle parts were severely degenerated, with a significantly increased MFF (Fig. 3B*, *Table 2). Except for rectus femoris, this proximo-distal gradient was observed in all lower limb muscles in sIBM. However, in a subset of the lower extremity muscles, a proximo-distal gradient was also found in PM-Mito and PM/ASyS/OLM (Fig. 3B, Suppl. Fig. 2), e.g., vastus medialis and flexor hallucis longus (PM-Mito), as well as vastus intermedius, vastus lateralis and sartorius (PM-Mito, PM/ASyS/OLM).

In contrast to the findings in lower extremities, the proximal muscles studied (gluteus medius and sacrospinalis) showed a significantly increased MFF in PM/ASyS/OLM and IMNM, while not being altered in sIBM and PM-Mito (Fig. 3B).

### Correlation of mean fat fraction and clinical parameters

**Fig. 4:**
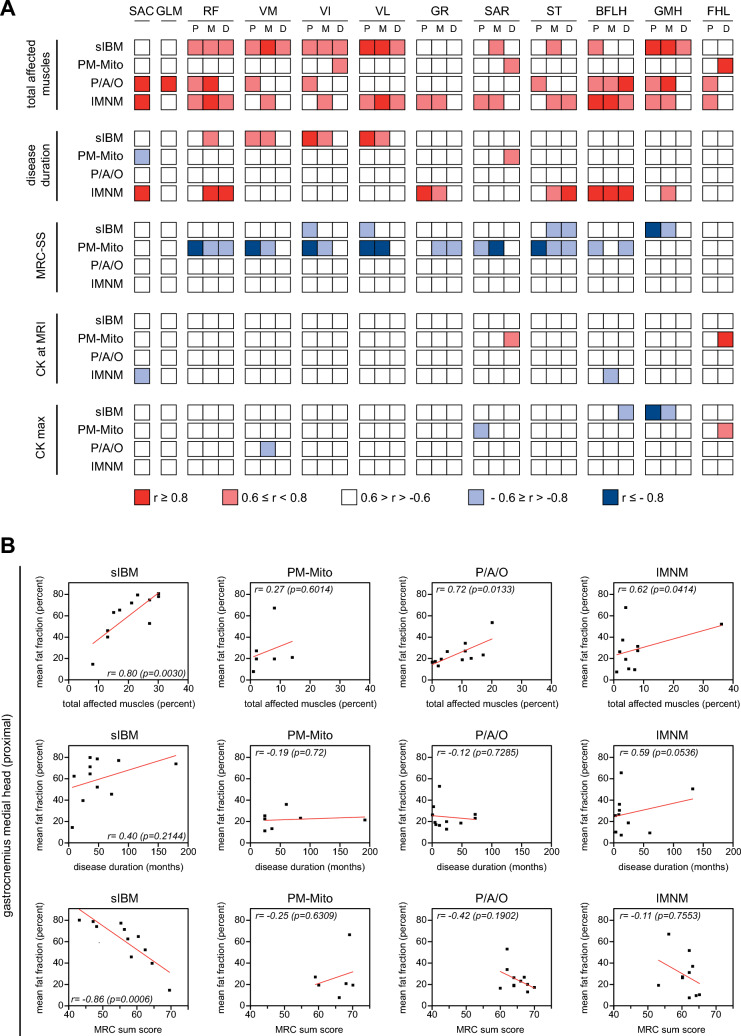
Correlation of mean fat fraction and clinical determinants of IIM progression. **A** Stratified correlation coefficient (Pearson) of mean fat fraction (MFF) correlated with clinical determinants of IIM progression. Except for PM-Mito, there is a relevant correlation of MFF with the total number of affected muscles (portion of muscles with Fischer grade ≥ 2) in all IIM subtypes. In sIBM, MFF of quadriceps femoris correlates with disease duration. A similar correlation is found in IMNM for semitendinosus, and long head of biceps femoris. There is a significant inverse correlation of modified MRC sum score (MRC-SS) and MFF in sIBM (semitendinosus, medial head of gastrocnemius), and PM-Mito (various muscles as indicated). No relevant correlations are evident between creatine kinase (CK) and MFF in all IIM subgroups. **B** Sample correlations of MFF and total affected muscles, disease duration, and modified MRC sum score, shown for the medial head of the gastrocnemius. Note the significant correlations of MFF with the total percentage of affected muscles and MRC sum score in sIBM. Additional examples can be found in Suppl. Fig. 3. *Abbreviations: SAC* Sacrospinalis, *GLM* Gluteus medius, *RF* rectus femoris, *VM* Vastus medialis, *VI* Vastus intermedius, *VL* Vastus lateralis, *GR* Gracilis, *SAR* Sartorius, *ST* Semitendinosus, *BFLH* Biceps femoris long head, *GMH* Gastrocnemius medial head, *FHL* Flexor hallucis longus, *P* proximal, *M* mid, *D* distal

The MFF of individual muscles correlated well with the total number of muscles affected on MRI. Whereas in PM-Mito only vastus intermedius, sartorius and flexor hallucis longus showed a respective correlation, a variety of muscles were identified in the other IIM subgroups, specific to each subgroup (Fig. 4A).

To address the clinical significance of quantitative MRI in IIM, MFF of the studied muscles were correlated with several clinical parameters used for disease monitoring (Fig. 4A). In sIBM, a relevant correlation of MFF and disease duration was observed in all muscles of the quadriceps femoris (Suppl. Fig. 3). Other predominantly affected muscles (e.g., medial head of the gastrocnemius) showed no correlation, nor did the proximo-distal gradient. In IMNM, a similar correlation between MFF and disease duration was seen for the long head of biceps femoris, gracilis, semitendinosus, medial head of gastrocnemius, rectus femoris, and sacrospinalis. In PM-Mito and PM/ASyS/OLM, there was no convincing correlation between disease duration and MFF.

Regarding the severity of the clinical phenotype, there was a significant inverse correlation between MFF and the modified MRC-SS for different muscles of sIBM and PM-Mito. In sIBM, such an inverse correlation was found in vastus intermedius and lateralis (proximal part), semitendinosus (mid and distal parts), and proximal as well as mid part of the medial head of gastrocnemius (Fig. 4B). For PM-Mito, a respective inverse correlation was seen for all quadriceps femoris muscles (especially proximal and mid parts), as well as sartorius, semitendinosus, and long head of biceps femoris. No correlation was found between MFF and clinical severity in PM/ASyS/OLM and IMNM.

Overall, there was no convincing correlation between CK values and MFF, both for maximum CK and CK at MRI.

### Treatment-related effects on different MRI parameters

Analysis of treatment effects appears difficult as more patients were treated (25 vs. 16), and untreated patients had shorter disease duration (total cohort 21.8 vs. 55.5 months, *p* = 0.033, Student's *t* test). To address this, analysis of covariance was performed including disease duration as covariate examining the different dependent variables (average MFF, average Fischer grade, average fraction of affected muscles, average fraction of edema-positive muscles) for the total cohort and each subgroup (sIBM, PM-Mito, PM/ASyS/OLM, IMNM). None of these analyses showed a statistically significant treatment effect on MRI changes. As subgroup analyses resulted in small group sizes, additional non-parametric Mann–Whitney *U* tests were calculated to compare groups (treated vs. non-treated). A statistically significant association was found only for mean MFF in sIBM (*p* = 0.017), but MFF in sIBM also correlated positively with disease duration (Spearman's rho = 0.685, *p* = 0.020). Thus, the significance of this effect remains doubtful (Suppl. Tab. 6).

## Discussion

The present study reports on one of the largest cohorts of patients with IIM studied with quantitative whole-body MRI to date. It is also the first study to directly address PM-Mito as a distinct IIM subtype.

In a first approach, muscular degeneration of 56 different muscles was semiquantitatively assessed to identify differences between subtypes of IIM, and to derive a disease-specific pattern of degeneration. While patients with sIBM showed a rather severe fatty replacement, modest changes were seen in most of the patients with PM-Mito, as well as in about half of those with PM/ASyS/OLM and three thirds of those with IMNM. This finding reflects the progressive, degenerative nature of sIBM and in contrast the treatable, potentially reversible muscular damage of other forms of IIM [45]. However, a relevant subset of patients with PM/ASyS/OLM and IMNM showed marked muscular damage. This phenomenon has previously been described in various forms of IIM, particularly in patients with IMNM [29]. The fact that fatty degeneration in IMNM was less common in the present study may partly rely on the disease duration at time of MRI, which was shorter in IMNM and PM/ASyS/OLM. However, the observed degenerative changes in both PM/ASyS/OLM and IMNM suggest a risk for irreversible muscular damage, emphasizing the need for early and sufficient immunosuppressive treatment.

**Fig. 5:**
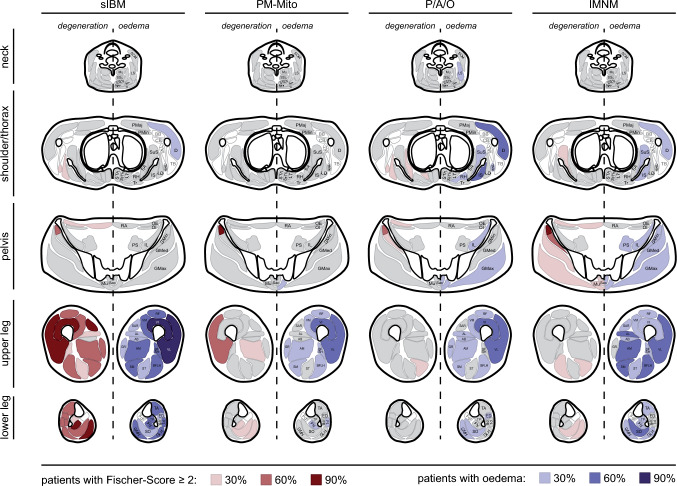
Schematic representation of muscular involvement in IIM subgroups based on semiquantitative assessment. Color graduation is based on the proportion of patients with fatty fibrous degeneration (right hemibody), and edema (left hemibody), respectively. Note the predominant affection of lower extremities in sIBM. In PM-Mito and PM/ASyS/OLM (P/A/O) there is an inconsistent and generally mild degeneration. If present, muscular degeneration tends to affect gluteal and paraspinal muscles in IMNM. While edema is mainly restricted to the lower extremities in sIBM and PM-Mito, proximal muscles (pelvis, trunk, shoulder and neck) are also affected in PM/ASyS/OLM and IMNM. *SCM* Sternocleidomastoideus*, LS* Levator scapulae*, Mu* Multifidus*, SSc/SSca* Semispinalis cervicis/capitis*, Spc/Spca* Splenius cervicis/capitis*, Tr* Trapezius*, PMaj/PMin* Pectoralis major/minor*, BB* Biceps brachii*, CB* Coracobrachialis*, TMi* Teres minor*, TMa* Teres major*, TB* Triceps brachii*, D* Deltoideus*, SuS* Subscapularis*, SA* Serratus anterior*, IS* Infraspinatus*, LD* Latissimus dorsi*, RH* Rhomboideus*, LT* Longissimus thoracis*, SsTh* Semispinalis thoracis*, STh* Spinalis thoracis*, RA* Rectus abdominis*, OE/OI* Obliquus externus*/*internus*, PS* Psoas*, IL* Iliacus*, GMin/GMed/GMax* Gluteus minimus/medius/maximus*, Sac* Sacrospinalis*, RF* Rectus femoris*, VL/VI/VM* Vastus lateralis*/*intermedius*/*medialis*, SAR* Sartorius*, GR* Gracilis*, AL/AB/AM* Adductor longus/brevis/magnus*, SM* Semimembranosus*, ST* Semitendinosus*, BFLH/BFSH* Biceps femoris long head/short head*, TA* Tibialis anterior*, ED* Extensor digitorum longus*, PB/PL* Peroneus brevis/longus*, TP* Tibialis posterior*, SO* Soleus, *Fdl* Flexor digitorum longus*, Fhl* Flexor hallucis longus*, GMH/GLH* Gastrocnemius medial head/lateral head

Regarding the disease-specific patterns of muscular degeneration, Fig. 5 shows a schematic representation of the summarized results. A consistent subset of muscles was found to be typically involved in sIBM. Predominant involvement of the lower extremities with early and typical affection of quadriceps femoris (particularly vastus lateralis and intermedius), as well as sartorius, medial head of gastrocnemius, and flexor hallucis longus was found. Apart from this core subset, most of the lower limb muscles appeared to be potentially involved in sIBM, with exception of adductors (especially adductor longus et brevis), semitendinosus, and short head of the biceps femoris. This is in good agreement with previous reports, describing a similar pattern of muscular affection, including inter-individual variability in patients with sIBM [33–36]. The lack of relevant upper extremity involvement is probably due to MRI acquisition procedures. The forearms, which are typically affected in sIBM and therefore relevant for differentiation from other IIM variants, are located at the edge of the field of view and therefore cannot be adequately assessed. In addition, the local resolution is relatively low and does not allow adequate discrimination of individual muscles. Other studies have shown variable MR morphological involvement of the upper extremities in sIBM [33].

In contrast to the consistent morphological changes observed in sIBM, no specific pattern of muscular degeneration was identified in PM-Mito and PM/ASyS/OLM. This may be partly due to the overall low prevalence of fatty involution in these subgroups, which may not allow the identification of congruent alterations. Furthermore, the individual response to immunosuppressive therapy with corresponding impact on degeneration and MR-morphology has to be considered. Similar influencing factors may also apply to IMNM. However, muscular degeneration appeared to be pronounced in some IMNM patients, with a comparable selectivity for gluteal muscles, hamstrings (long head of biceps femoris and semimembranosus), and posterior compartment of the lower legs (medial head of gastrocnemius, soleus). Similar changes have been reported previously [29]. However, Pinal-Fernandez et al. also observed a relevant involvement of the adductors, not observed in this study.

With regard to intramuscular edema, the pattern of distribution was mainly restricted to the muscles identified as being subject of muscular degeneration. Accordingly, gluteal and axial muscles as well as upper extremities were more severely affected in PM/ASyS/OLM and IMNM, while predominant degeneration of the lower extremities was observed in sIBM. Edema was generally less pronounced in PM-Mito. These results might argue for a direct impact of muscular inflammation in the pathophysiology of muscular degeneration in IIM, as suggested by previous reports [46, 47]. Again, there was a high inter-individual variation, both within specific IIM subgroups and between the subgroups. This may be related to the aforementioned clinical heterogeneity regarding disease duration and immunosuppressive treatment.

Several previous studies identified asymmetry as a typical feature of sIBM in distinction to other forms of IIM [32, 33, 36]. Asymmetry in both muscular degeneration and edema in sIBM was confirmed in this study. However, a similar degree of asymmetry was observed in other forms of IIM, questioning the specificity of this feature. This is in contrast to the clinical impression of a typically symmetrical involvement in PM-Mito, PM/ASyS/OLM and IMNM. The reason for this phenomenon remains elusive. Subclinical differences only detected by MRI may be discussed.

To further evaluate the role of muscular degeneration in disease delineation and to address the role of quantitative MRI as a biomarker of disease severity, mean fat fraction (MFF) of selected muscles was analyzed. Consistent with the semi-quantitative findings, MFF was higher in sIBM compared to all other IIMs. These differences were particularly evident in the distal parts of the muscles, while the MFF of the proximal parts was not significantly altered. Thus, a proximo-distal gradient of muscular degeneration appeared to be a typical feature of sIBM in nearly all lower limb muscles examined. This aligns with previous reports [34, 37]. In contrast, most of the PM-Mito patients did not display similar findings, even after several years of disease activity. Therefore, the results of the present study argue against PM-Mito as an sIBM precursor in general, as suggested by some authors [11–15]. However, a small subset of PM-Mito and PM/ASyS/OLM patients showed MRI-features that were observed in sIBM, especially with regard to the proximo-distal gradient. These patients may indeed be in an oligosymptomatic stage of sIBM, lacking definite clinical or histological characteristics. In these cases, quantitative MRI may be a novel clinical tool to identify these patients at an early stage, facilitating the application of disease-specific therapies in clinical trials and routine practice. Furthermore, this interesting overlap may be indicative of a spectrum disorder ranging from PM/OLM and PM-Mito toward sIBM [16, 45, 48]. This is of particular importance, especially regarding immunosuppressive therapy. Based on the evidence gained, there are no arguments that should generally limit the use of immunosuppressive therapy in patients with PM-Mito. However, the approach presented in this study appears to be highly operational. The integrated use of clinical, serological and muscle biopsy information, together with MRI examination, is mandatory to further facilitate the delineation of IIM subgroups and to allow an informed decision regarding the immunosuppressive strategy.

In contrast to the findings in the lower extremities, there was a relevantly increased MFF in gluteal and paraspinal muscles in PM/ASyS/OLM and IMNM, whereas corresponding changes were not seen in sIBM and PM-Mito. This observation may reflect the predominant proximal and axial involvement in PM/ASyS/OLM and IMNM, as demonstrated for IMNM in a previous study [49].

To address the suitability of quantitative MRI-imaging as surrogate for clinical severity, MFF was correlated with several clinical and radiological parameters. For several muscles, there was a significant correlation between MFF and the total number of affected muscles in sIBM (quadriceps femoris, sartorius, semitendinosus, long head of biceps femoris, medial head of gastrocnemius), PM/ASyS/OLM (sacrospinalis, gluteus medius, rectus femoris, vastus medialis, vastus intermedius, semitendinosus, long head of biceps femoris, flexor hallucis longus, medial head of gastrocnemius), and IMNM (all muscles studied except gluteus medius). Therefore, MFF of these muscles may serve as a surrogate for the overall burden of degenerative changes. Focused quantitative assessment of these muscles may reduce resources, both in terms of acquisition and evaluation. No such correlation was found in PM-Mito.

An inverse correlation was observed when comparing the disease severity as measured by a modified MRC sum score and MFF for selected muscles for sIBM (medial head of gastrocnemius, Fig. 4B), and PM-Mito (members of quadriceps femoris and hamstrings). Thus, the results of this study suggest quantitative MRI measures as a potential clinical tool to monitor disease progression in a subset of IIM patients (e.g., sIBM and PM-Mito). In this context, it seems contradictory that no significant treatment effects were observed on the MRI parameter studied. However, there was a significant difference in the number of treated and untreated patients. In addition, treated patients had a significantly longer disease duration. Both may be relevant confounding factors. In addition, a relevant inter-individual variability in MR morphological response to treatment must be considered, which would be best captured in sequential examinations. Therefore, longitudinal analyses in larger cohorts are mandatory to further investigate the suitability of MFF as a potential biomarker in specific IIM subtypes. In this regard, a recent study in sIBM has shown promising results [50]. Notably, most of the muscles identified as typically affected in sIBM did not show a significant correlation with clinical severity. This may be explained by an early and exaggerated involvement of these muscles that does not reflect the overall clinical progression. There was no correlation between clinical affection and MFF in PM/ASyS/OLM and IMNM. Again, this could be seen as an argument for both the heterogeneity of these patients and the potentially reversible muscular damage not necessarily translating into persistent degeneration.

This study has several obvious limitations. Although the present study is one of the largest quantitative whole-body analyses in terms of the total number of studied patients, the size of the individual subgroups (especially PM-Mito) is comparatively small, impeding data interpretation. Another relevant issue is the retrospective nature of the analysis, resulting in a relevant heterogeneity in terms of disease duration, immunosuppressive treatment, and availability of clinical information (including serological status). This applies in particular to the PM/ASyS/OLM subgroup that—from a clinical standpoint—shows marked differences in clinical and histological appearance. However, patients with PM/ASyS/OLM in this study had similar MR morphological features, and separation would have resulted in even smaller group sizes. In this respect, the PM/ASyS/OLM subgroup in this study can be considered as a combined disease-control group distinct from sIBM. In addition, clinical scores for disease severity were not consequently captured. Hence, the MRC-SS was the only score suitable for retrospective derivation from the available clinical data. However, this score was not consequently evaluated in the context of IIM. Furthermore, some of the MRI parameters (Fischer grading and edema evaluation) used in this study are semiquantitative measures and therefore may be subject to an inter-observer bias. To our knowledge, there is no specific study on the inter-observer reproducibility of the Fischer score. However, based on findings for other semi-quantitative radiological parameters, a relevant bias must be considered [51]. The MFF as a quantitative measure has been shown to have high reproducibility when used with manual muscle segmentation based on anatomical landmarks, as used in this study [52].

In summary, the results of this study delineate sIBM patients from other forms of IIM, both in semiquantitative and quantitative approaches. Of particular interest, patients with PM-Mito did not consistently show alterations observed in sIBM, even in the sensitive quantitative measures applied. This argues against the hypothesis that PM-Mito is a slowly progressive variant of sIBM per se, but favors a spectrum disorder including PM/OLM, PM-Mito, and sIBM. Alternatively, the mitochondrial pathology in PM-Mito may also be an epiphenomenon, not pointing to a specific disease category. As techniques for early detection of sIBM patients have been identified as a major issue for targeted inclusion in clinical trials [53], quantitative MRI might sufficiently add to the diagnostic toolbox in IIM. This also applies with regard to objective measures of disease progression and severity.

### Supplementary Information

Below is the link to the electronic supplementary material.

Suppl. Fig. 1 | Asymmetry of muscular affection. A Proportion of patients with asymmetric involvement of fatty fibrous degeneration (as indicated by Fischer grade) and edema. Asymmetry is frequently observed in all IIM subgroups, both in terms of muscular degeneration and edema. B+C Quantification of affected muscles with asymmetry of muscular degeneration (B), and edema (C). Middle dash represents the average proportion, while whiskers indicate one standard deviation, and dots show individual patient's values. There are no differences in the grade of asymmetry between different IIM subgroups. Abbreviations: Sternocleidomast. Sternocleidomastoideus, Spl. Splenius, cap. capitis, cerv. cervicis, scap. scapulae, Semisp. Semispinalis, maj. major, min. minor, thor. thoracis, dors. dorsi/dorsal, ant. anterior, abd. abdominis, int. internus, ext. externus, max. maximus, min. minimus, med. medius/medialis, Add. Adductor, long. longus/longum, brev. brevis, magn. magnus, fasc. lat. fasciae latae, fem. femoris, intermed. intermedius, lat. lateralis, s.h. short head, l.h. long head, post. posterior, Ext. digit. Extensor digitorum, Gastrocn. Gastrocnemius, m.h. medial head, l.h. lateral head, Flex. hall. Flexor hallucis longus. Supplementary file1 (EPS 1922 KB)

Suppl. Fig. 2 | Quantitative assessment of muscular fat fraction in IIM subgroups (continued). MFF of selected muscles in proximal, mid, and distal parts of the respective muscle (left panels), and determination of the distality ratio (MFF of distal versus proximal parts, right panel). Middle dash represents the average MFF, while whiskers indicate one standard deviation, and dots show individual patient's values. Abbreviations: m.h. medial head. Supplementary file2 (EPS 2111 KB)

Suppl. Fig. 3 | Correlation of mean fat fraction and clinical determinants of IIM progression (continued) - Sample correlations of MFF and total affected muscles, disease duration, and modified MRC sum score in selected muscles. Supplementary file3 (EPS 3269 KB)

Supplementary file4 (XLSX 12 KB)

Supplementary file5 (XLSX 13 KB)

Supplementary file6 (XLSX 43 KB)

Supplementary file7 (XLSX 35 KB)

Supplementary file8 (XLSX 27 KB)

Supplementary file9 (XLSX 36 KB)

Supplementary file10 (XLSX 29 KB)

## Data Availability

All data used for this study are available in de-identified form in the Electronic Supplementary Material section.
